# Testicular Function After Immune‐Checkpoint Inhibitors Treatment

**DOI:** 10.1111/cen.70001

**Published:** 2025-07-14

**Authors:** Andrea Boutros, Amanda Idan, Sue Sleiman, Feyrous Bacha, Ting Zhang, Veena Jayadev, Alexander M. Menzies, Georgina V. Long, David J. Handelsman

**Affiliations:** ^1^ Melanoma Institute Australia The University of Sydney Sydney New South Wales Australia; ^2^ U.O. Clinica di Oncologia Medica IRCCS Ospedale Policlinico San Martino University of Genoa Genoa Italy; ^3^ Andrology Department Concord Hospital Concord New South Wales Australia; ^4^ Clinical Andrology Laboratory Concord Hospital Concord New South Wales Australia; ^5^ Faculty of Medicine and Health The University of Sydney Sydney New South Wales Australia; ^6^ Royal North Shore Hospital, Northern Sydney Local Health District Sydney New South Wales Australia; ^7^ Mater Hospital North Sydney New South Wales Australia; ^8^ ANZAC Research Institute The University of Sydney Sydney New South Wales Australia

**Keywords:** azoospermia, cancer survivorship, immune checkpoint inhibitors, male fertility, melanoma, reproductive toxicity, sperm cryopreservation, spermatogenesis

## Abstract

**Objective:**

To investigate the effects of immune‐checkpoint inhibitors (ICIs) on spermatogenesis and testicular endocrine function in reproductive‐age men with melanoma.

**Design:**

Prospective, mixed longitudinal and cross‐sectional cohort study.

**Patients:**

Twenty‐nine men aged 19–46 years undergoing ICI therapy for melanoma at two Australian centres between 2019 and 2024. Three patients were excluded due to subsequent gonadotoxic therapies. The remaining 26 were evaluable.

**Measurements:**

Semen analysis and serum hormone levels (FSH, LH, testosterone) were assessed at baseline and after ICI exposure. Patients with prior cytotoxic chemotherapy or pelvic radiotherapy were excluded.

**Results:**

Among 26 evaluable patients, one man developed persistent azoospermia with marked serum FSH elevation. Overall, median total sperm output per ejaculate declined by 34% (193–127 million per ejaculate) and sperm concentration by 30% (61–43 million/mL), neither statistically significant before or after excluding the case of presumed autoimmune orchitis. On treatment, serum LH and testosterone remained stable while the increase in serum FSH (*p* = 0.04) was no longer significant after excluding the single man with auto‐immune orchitis.

**Conclusions:**

ICI treatment may be associated with minimal spermatogenic dysfunction, as reflected by modest reductions in sperm output and increased serum FSH levels, despite preserved serum LH and testosterone concentrations, but mostly due to a rare (4% prevalence) case of autoimmune orchitis. The unpredictability of these effects supports routine fertility preservation counselling in young men before ICI initiation.

## 1 Introduction

Immune‐checkpoint inhibitors (ICI) have become one of the most widely used therapies across the cancer field [1, 2, 3]. ICIs revolutionised the treatment of melanoma, a cancer once considered terminal in its advanced stages, leading to long‐term benefit in nearly half of patients, and a potential cure [4, 5]. These advances paved the way for broader application of ICIs to include earlier stage disease in the neoadjuvant and adjuvant settings, including as early as stage IIB [6, 7, 8, 9, 10, 11, 12].

Importantly, melanoma often affects patients of reproductive age, raising concerns about the impact of cancer treatment on fertility, particularly considering prolonged survival rates [13].

While the long‐term endocrine toxicities of ICIs, such as hypothyroidism, hypopituitarism, and adrenal insufficiency, are well‐known, occurring in approximately 1%–15% of patients depending on the treatment regimen, the potential gonadal toxicities of ICIs remain underexplored [14, 15]. A previous study of 34 men (including 16 with melanoma) before and during non‐cytotoxic and immunotherapy treatments, found a 50% reduction in sperm production with increased FSH and decreased inhibin B [16]. A preclinical study in female mice has demonstrated that ICIs can cause immune‐related ovarian damage, reducing both the number and size of oocytes with increased immune cell infiltration possibly mediated by increased inflammatory cytokines [17]. In contrast, data on ICI‐related male hypogonadism are scarce, limited to case reports and small studies [14, 18, 19, 20]. For instance, a cross‐sectional study showed that four out of 22 male patients treated with ICIs had abnormal semen analyses, including cases of oligo‐ and azoospermia, although confounding factors such as prior radiotherapy or alcohol abuse were present and few data were available on pre‐treatment sperm output [18].

The dramatic improvement in survival outcomes brought about by ICIs necessitates a broader focus on survivorship and quality of life. With more patients achieving durable remissions, particularly in younger cohorts, the long‐term effects of treatment on reproductive health, endocrine function, and overall quality of life must be systematically addressed. Understanding these impacts is vital not only for optimising cancer care but also for enabling survivors to make informed decisions about family planning and future fertility [21, 22].

The present study aims to expand knowledge about the gonadal toxicities associated with melanoma treatments, and in particular ICIs. Specifically, we seek to clarify the incidence and clinical implications of ICI‐induced impact on testicular exocrine and endocrine function, providing insights to guide fertility preservation and survivorship care.

## 2 Methods

### 2.1 Study Design and Population

This prospective observational study expanded our previous findings on the impact of non‐cytotoxic drug treatments on reproductive function with specific focus on men with melanoma receiving ICI therapy [16]. We evaluated changes in sperm output and testicular function following systemic treatments recruiting men of reproductive age with melanoma. The primary endpoint was the difference in sperm output before and during or after ICI treatment. The study was conducted at two Australian centres: the Andrology Department of Concord Hospital and the Melanoma Institute Australia, Sydney, New South Wales. Male patients aged 18–65 years with melanoma requiring systemic therapy were eligible for inclusion if they had not previously received potentially gonadotoxic treatments with cytotoxic chemotherapy or pelvic radiotherapy. Patients with baseline oligo/azoospermia of any known cause were excluded. The study, with the written informed consent form, was approved by the Sydney Local Health District Human Ethics Committee (Concord Hospital) and was conducted in accordance with the Declaration of Helsinki.

### 2.2 Study Procedures

Participants attending for sperm cryopreservation underwent andrological assessments comprising a medical and reproductive history with a physical examination (including testicular volume) as standard clinical care for men with cancer scheduled to undergo potentially gonadotoxic treatments [23, 24]. During regular follow‐up visits, the medical and reproductive history was updated with semen analysis and hormone measurements to evaluate need for ongoing cryostorage. Blood and semen samples were obtained at baseline and during follow‐up visits, continuing after treatment completion as part of routine post‐cryostorage clinical care. Participants who did not have pre‐treatment sperm cryostorage but were undergoing ICI treatments underwent similar evaluations, including medical history, physical examination, and collection of blood and semen samples.

### 2.3 Laboratory Analyses

Semen parameters evaluated included semen volume, sperm concentration, motility, and morphology according to the WHO Semen Manual 6th edition [25] in the Clinical Andrology laboratory, accredited regularly for decades implementing ISO 15189 criteria by the National Association of Testing Authorities (NATA), Australia's national agency for certification of clinical testing laboratories. For these men whose fertility was mostly unknown and who were not seeking fertility, we utilised reference values for unselected men from the WHO normative study whereby oligozoospermia was defined as < 20 million sperm per ejaculate [26]. The primary study endpoint, sperm output, was the product of semen volume and sperm concentration. Blood samples were collected in the morning where feasible and analysed for reproductive hormones using commercial immunoassays to measure serum luteinizing hormone (LH), follicle‐stimulating hormone (FSH), testosterone, and sex hormone‐binding globulin (SHBG) [27].

### 2.4 Statistical Analysis

Changes in sperm parameters and reproductive hormone levels over time were analysed using linear mixed‐effects models for repeated measures with an autoregressive covariance structure, which features no requirement for complete data or fixed schedule of visits. The pre‐treatment baseline data were from sperm cryostorage samples, usually involving three samples collected within a week. Follow‐up visits were as determined by their medical care and not in any fixed timing. Data were presented as mean ± standard error of the mean (SEM) for normally distributed variables or as median [interquartile range] (IQR) for non‐normally distributed data. Skewed sperm concentration and output were cube‐root transformed to approximate a normal distribution before analysis [28]. Adjustments were made for potential confounders, including age, body mass index, treatment type, lifestyle factors and autoimmunity. Statistical significance was defined as *p* < 0.05.

## 3 Results

### 3.1 Cohort Characteristics

**Figure 1:**
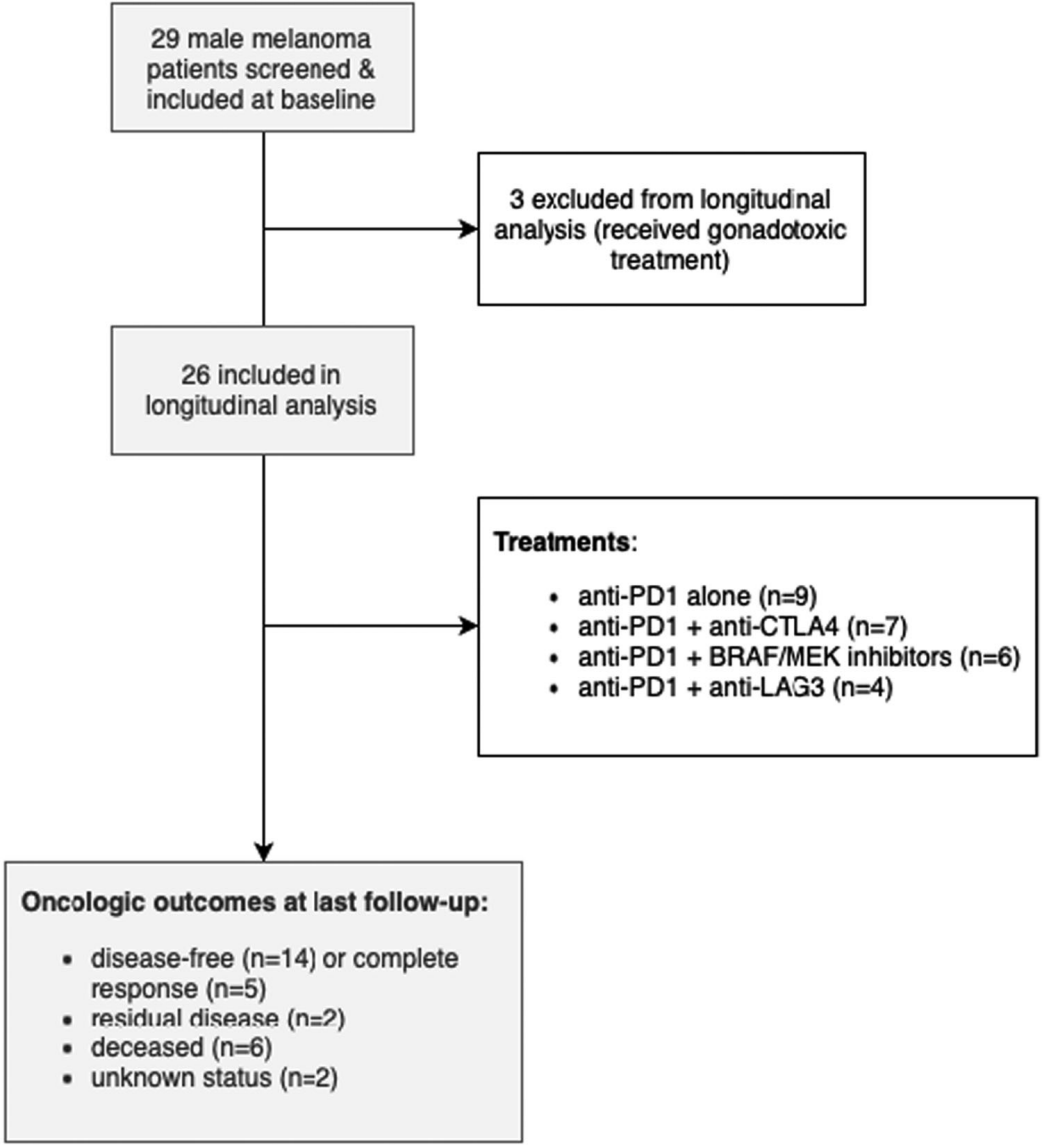
Patients' disposition. A total of 29 male melanoma patients were enroled and assessed at baseline. Three were excluded from the longitudinal analysis due to subsequent gonadotoxic treatments. The remaining 26 patients received immune checkpoint inhibitor (ICI) therapies, including anti‐PD1 alone or in combination with anti‐CTLA4, anti‐LAG3, or BRAF/MEK inhibitors.

We evaluated 29 men aged 19–46 years of age with melanoma over 91 visits, with varying follow‐up intensity (one visit: 29 patients; two: 27; three: 20; four: 8; five or more: 7) (Figure 1). The mean age was 32 ± 1 years, height 183 ± 1 cm, weight 87.7 ± 3.3 kg, BMI of 26.2 ± 0.8 kg/m^2^, and BSA 2.11 ± 0.04 m^2^. Most (75%) were married with prior paternity established in 28%, and none had cryptorchidism. Most (81%) never smoked and 19% past smokers, with 62% having light or no alcohol intake but no heavy drinkers (Table 1).

**Table 1:** Baseline reproductive functions of patients.

Variable	Number	Mean ± SEM	Reference Values
Age (years)	29	32 ± 1	
Mean testis volume (mL)	15	27 ± 1	
Semen volume (mL)	29	3.6 ± 0.4	> 1.2
Sperm concentration (million/mL)	29	83 (35, 126)*	> 9
Sperm output (million)	29	172 (81, 546)*	> 20
Motility (%)	29	42 ± 4	> 26
Sperm Morphology (normal %)	29	7.0 ± 0.8	> 3.5
Oligozoospermic samples			
Pretreatment	26	4 (15%)	
On‐treatment	54	4 (7%)	
Serum testosterone (nmol/L)	27	17.1 ± 1.4	7.4–28.0
Serum LH (IU/L)	27	4.5 ± 0.4	5.1–18.7
Serum FSH (IU/L)	27	4.4 ± 0.5	1.3–12.0

*Note:* Data are presented as mean ± standard error of the mean (SEM) or as median (interquartile range, *), where specified. Reference values for semen variables are from WHO and serum testosterone, LH and FSH are from the Raine Birth Cohort study [25, 29]. Body mass index is calculated as weight/height^2^, and body surface area (BSA) according to the Gehan‐George formula [30].

At baseline, 22 (76%) presented with stage II/III (‘early’) melanoma, while 7 (24%) had stage IV (‘advanced’) disease. Treatment regimens included anti‐PD1 in 24 patients (nine alone, seven with anti‐CTLA4, four with anti‐LAG3, and six with BRAF/MEK inhibitors).

After a follow‐up time of a median of 2.8 years (IQR 1.7, 4.5 years) at the time of analysis, 21 patients (72%) were alive, with 19 disease‐free (including five with complete response), and two with residual disease. Six patients (21%) had died, and follow‐up data were unavailable for two patients (7%). Regarding treatment‐related adverse events, five patients (17%) developed endocrine immune‐related adverse events: three cases of hypothyroidism and two of hypopituitarism.

### 3.2 Fertility and Sperm Output

Twenty‐nine men were included in the study. Three men were included only in the baseline pre‐treatment evaluation because of subsequent gonadotoxic treatments (two had inguinal nodal basin radiotherapy, two had chemotherapy and one had both treatments). All longitudinal outcome analyses involved the remaining 26 men who undertook pre‐treatment sperm cryostorage.

At baseline, one man had azoospermia and three had moderate oligozoospermia (10–20 million sperm per ejaculate) with two of the oligozoospermic men having had prior paternity. On treatment, three men had their cryostored sperm transferred to a fertility clinic with two producing pregnancies by natural conception without needing to use cryostored sperm. One man still has cryostored embryos created before diagnosis for nonmedical reasons but had not yet used them to induce pregnancy.

**Figure 2:**
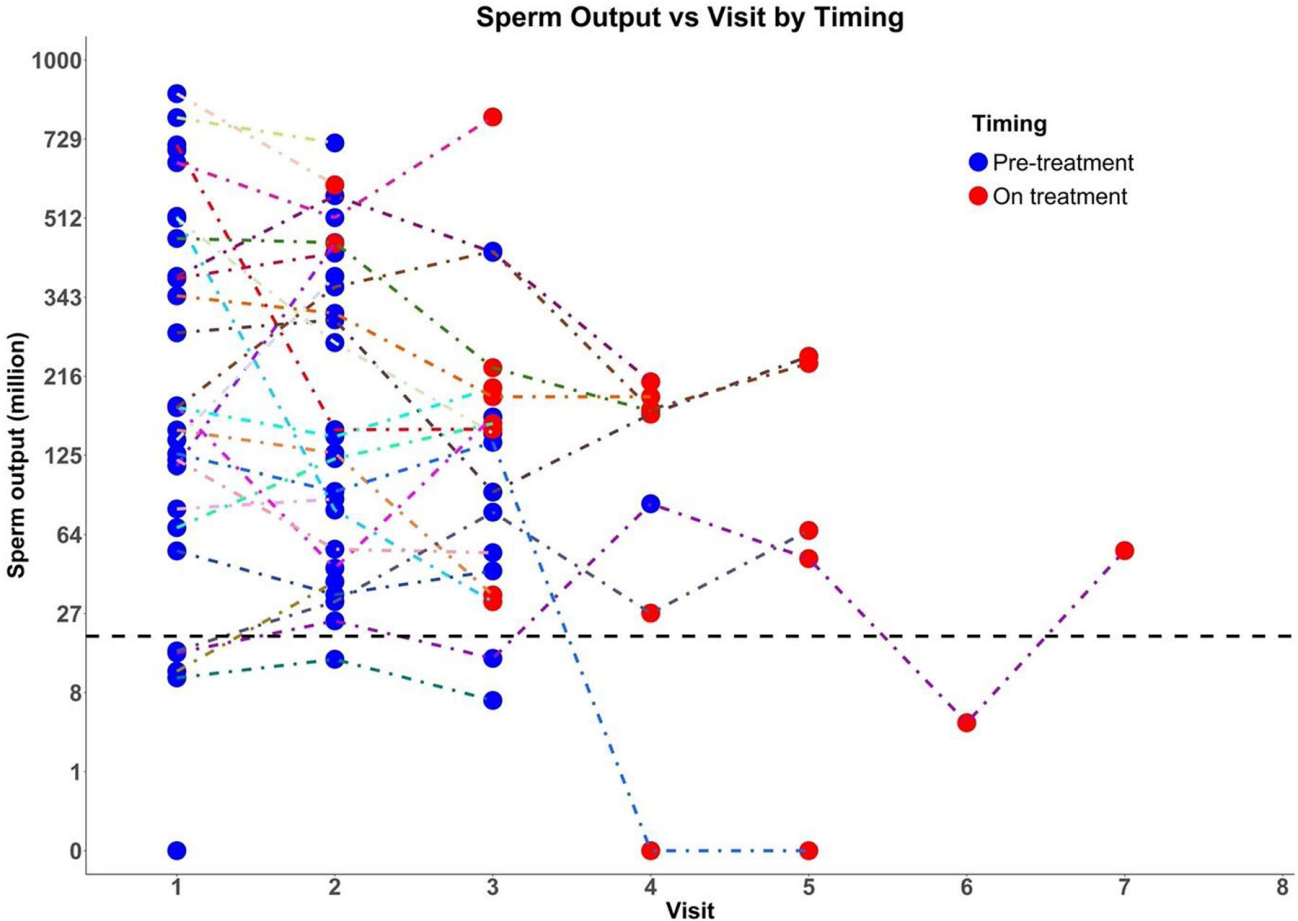
Longitudinal analysis of sperm output across clinical visits, comparing pre‐treatment (blue dots, *n* = 29) and on‐treatment (red dots, *n* = 26) measurements. The y‐axis represents sperm output in millions using a cubic scale to accommodate variability, while the x‐axis displays the sequential visit number (1–7). The visits are not on a fixed time schedule. Pre‐treatment up to three sperm cryostorage samples are usually collected within a week but on‐treatment visits depend on the clinical management schedule without fixed time intervals. Dotted lines connect individual subject measurements over time, highlighting intra‐subject variability. The thick horizontal dashed line represents the threshold distinguishing normal from low sperm output. The analysis shows a general trend of relatively stable sperm output from pre‐treatment to on‐treatment phases, with considerable variability observed among subjects and one man who develops persistent azoospermia.

Sperm output per ejaculate decreased by 34% from a median of 193 million (pre‐treatment) to 127 million (on treatment) (*p* = 0.053) (Figure 2). Similarly, sperm concentration declined by 30% from 61 million/mL to 43 million/mL (*p* = 0.075). There were no significant changes in sperm motility (43 ± 2 vs. 43 ± 4%, *p* = 0.94) or morphology (7.4 ± 0.5 vs. 6.5 ± 0.8%. *p* = 0.31). Covariate analysis showed that sperm output during or after ICI treatment was not significantly different according to age, mean testis volume, marital or fertility status, smoking or alcohol consumption or autoimmunity.

One 28‐year‐old man with normal pre‐treatment sperm output and reproductive hormone profile developed azoospermia on treatment. He underwent 36 days of neoadjuvant anti‐PD1 and BRAF/MEK inhibitor treatment achieving a pathologic complete response, followed by 1 year of postsurgical anti‐PD1 monotherapy. One year after starting treatment, he demonstrated azoospermia with elevated serum FSH, while serum LH and testosterone remained normal, and azoospermia still persisting 3 years post‐treatment. His autoantibody screen was positive for mid‐body nuclear antibody (titre 1:320) and myositis‐specific antibody (MSA, title 1:80) but negative for IgG and IgA sperm antibodies, transglutaminase, adrenal, extractable nuclear antigen, gastric parietal cell, intrinsic factor, anti‐neutrophil and neutrophil cytoplasmic antibodies. No other medications were administered, and no immune‐related endocrinopathies were identified. None of the participants including this man developed testicular pain during treatment.

The marginally significant reduction in sperm output was largely attributable to the findings in that man because, in a sensitivity analysis excluding his data, there was no longer a significant reduction in sperm output (*p* = 0.39), sperm concentration (0.09) or serum FSH (0.82).

### 3.3 Serum Reproductive Hormones

Compared with pretreatment, serum FSH was significantly increased (4.7 ± 0.8 vs. 6.7 ± 0.9 IU/L, *p* = 0.04) whereas serum LH (*p* = 0.14), testosterone (*p* = 0.18) and SHBG (*p* = 0.71) remained unchanged by ICI treatments. The increase in serum FSH was due to the one man who developed azoospermia because the on‐treatment serum FSH was no longer significantly different from pretreatment if his data was excluded.

## 4 Discussion

This is the first prospective pilot study on men, free from prior gonadotoxic treatments, to investigate the impact of ICI treatment on human testicular function. Our findings demonstrate minimal reductions in sperm output during treatment, accompanied by a significantly increased serum FSH reflecting disruptions in testicular function, predominantly spermatogenesis but largely due to the impact of one man who developed apparent autoimmune orchitis.

Although most ICI‐treated men continued to have minimally diminished sperm output, one man developed persistent azoospermia during ICI‐treatment with no other apparent cause (Figure 2). Importantly, this study was conducted in a cohort of relatively young, healthy patients with mainly early‐stage melanoma, many of whom had no comorbidities or prior medications. This minimises confounding factors, strengthening the association between ICIs and observed reproductive changes. These observations of rare but unpredictable autoimmune orchitis highlight the need for proactive fertility preservation strategies, notably sperm cryostorage before treatment, and for a more comprehensive understanding of the gonadal toxicities associated with ICIs.

The observed reduction in sperm output (34%) and in sperm concentration (30%) was dominated by one man who had normal sperm output and reproductive hormones before treatment but then developed persistent on treatment for no other identified cause. Similarly, it aligns with preclinical evidence suggesting ICI treatment causes immune‐mediated testicular damage [31, 32]. None of the participants reported testicular pain during the study so the present findings suggest that a painless, subclinical form of autoimmune orchitis may occur in a small subset (< 5%) of ICI‐treated men. The concurrent increases in serum FSH with preserved serum LH and testosterone levels represent a key finding that identifies the nature of ICI impact on male reproductive function indicating a direct effect on the testis. The stability of serum LH and testosterone production suggests that overall gonadal endocrine function remains largely intact. Nevertheless, the impact on the testis indicates that overt Leydig cell failure and androgen deficiency may occur in the future when ageing effects are superimposed on post‐ICI treatment effects. Hence long‐term surveillance of testicular endocrine function by monitoring serum testosterone with LH and FSH together with awareness of possible androgen deficiency symptoms is required. The elevated serum FSH coupled with modest decline in sperm output and motility, reflecting a greater effect on seminiferous tubules and spermatogenesis, indicate that fertility impairment is a more immediate concern than longer‐term hormonal dysfunction. Long‐term surveillance of serum FSH may also indicate recovering spermatogenesis, if hypophysitis is excluded.

The present findings add to the wider issue of ICI effects on gonadal function to those already described in small subsets of men who experience ICI‐induced hypogonadism as either primary hypogonadism, due to orchitis, or secondary hypogonadism due to hypophysitis [14]. The present data also extends reports of ICI‐induced orchitis, reported in small subsets of ICI‐treated men, to a wider but less severe impact on most ICI‐treated men. Nevertheless, the present findings suggest that ICI treatments have a substantially lower impact overall on testicular function compared with traditional gonadotoxic cancer treatment, such as cytotoxic chemotherapy and radiotherapy, which typically cause severe and often irreversible damage to spermatogenesis [23, 24]. However, the unpredictable occurrence of auto‐immune orchitis with a prevalence of about 4% indicates the ongoing need for fertility counselling and sperm cryostorage before ICI treatment for melanoma.

These findings are particularly relevant given the mean age of 32 years in our cohort, representing an age for whom fertility preservation is a critical quality‐of‐life consideration [31]. With melanoma frequently diagnosed in reproductive‐age individuals and the expanding indications for ICIs across cancer, understanding and addressing fertility‐related concerns become increasingly important in comprehensive cancer care.

The blood‐testis barrier (BTB) typically provides an immune privileged site for developing germ cells, protecting spermatogenesis from systemic immune responses. For example, an intact BTB prevents bone marrow stem cells from colonising the testicular germinal epithelium, a biologically plausible possibility based on experimental germ cell transplantation, which could otherwise lead to men having donor genotype sperm after bone marrow transplantation [32, 33, 34]. However, our current findings suggest that ICI therapy may disrupt this protective mechanism, which could be particularly important if ICI‐treated men later require bone marrow transplantation.

The observed reproductive changes may result from increased inflammatory cytokines and immune cell infiltration disrupting the BTB integrity, as supported by preclinical studies [17]. This mechanism differs from the ovarian effects of ICIs, where the absence of a comparable immune barrier may result in direct follicular damage through immune‐mediated mechanisms [17]. Similarly, the responsiveness of melanoma brain metastases to ICIs and BRAF‐targeted therapy challenges traditional assumptions about the impermeability of the blood‐brain barrier [5, 35, 36].

Our findings expand the limited existing evidence regarding ICI‐associated male gonadal toxicity. Previous reports, including case studies and small cross‐sectional analyses, have described cases of oligospermia and azoospermia in ICI‐treated patients [14, 18]. In addition, a post‐mortem study of men who died from melanoma may have been confounded by terminal treatments, which likely had a greater impact on the testis than the pre‐mortem effects of ICI therapy [37, 38]. By focusing on treatment‐naïve patients and excluding those with prior gonadotoxic exposures, our study provides more definitive evidence linking ICI therapy to testicular dysfunction. Moreover, the hormonal changes we observed support a primary testicular effect, primarily on spermatogenesis and possibly via inducing an autoimmune orchitis.

Several limitations must be considered when interpreting our findings. First, the relatively small sample size reduces the generalisability of our results, although the longitudinal study design and the use of robust statistical methods enhance the reliability of the conclusions drawn. Additionally, while we excluded patients with prior gonadotoxic treatments, baseline reproductive health could still have been affected by unmeasured confounding factors. Moreover, the impact of BRAF/MEK inhibitors and other immunotherapy agents remains largely unclear with findings confined to animal data, in vitro studies and single case reports [19, 20, 39, 40]. As our study focused primarily on ICI effects as it was too small to dissect effects of anti‐PD1 with and without additional drugs. However, given the use of these targeted therapies in melanoma treatment even in the adjuvant setting, their potential reproductive effects warrant dedicated investigation. Finally, the absence of extended follow‐up data limits our ability to evaluate the potential long‐term reversibility of the observed changes in reproductive parameters after treatment completion.

These findings underscore the importance of reproductive health counselling in male melanoma patients undergoing ICI therapy, with a specific focus on fertility preservation by sperm cryostorage as insurance against spermatogenic damage that may prevent timely paternity. While the preserved testosterone levels are reassuring in the short‐term regarding overall testicular endocrine function, the documented effects on sperm parameters highlight the critical need for fertility preservation discussions before treatment initiation as well as long‐term follow‐up to identify long‐term posttreatment androgen deficiency. This distinction is important for patient counselling, as it suggests that while sexual function and secondary male characteristics are likely to be maintained in the patient's immediate future, fertility may be significantly impaired in some men and, in the long‐term, potentially androgen status.

The assessment of gonadal function in reproductive‐age patients receiving ICIs requires systematic monitoring. Given the demonstrated impact of ICIs on spermatogenesis, sperm cryostorage should be recommended to all men who have not completed their families before initiating immunotherapy [13]. This preventive approach is particularly crucial considering that fertility impairment may occur early in the treatment course and could be irreversible. The timing of fertility preservation is critical, as it should be performed before any exposure to ICIs to ensure optimal sperm quality.

Additionally, medical oncologists should actively enquire about symptoms of hypogonadism, including changes in libido and sexual function, as patients may not spontaneously report these concerns. This is particularly relevant given the expanding use of ICIs in both advanced disease and adjuvant settings, where quality of life considerations becomes increasingly important. The unique characteristics of testicular and ovarian immune responses suggest the need for sex‐specific approaches to fertility preservation in melanoma patients.

## 5 Conclusions

Our study provides the first prospective, preliminary, evidence on the impact of ICIs therapy for melanoma on testicular function in men, demonstrating overall modest effects on sperm output and function with raised serum FSH due to one man developing severe spermatogenic damage (azoospermia) due to presumed autoimmune orchitis. The findings of an unpredictable risk of autoimmune orchitis with a prevalence of < 5% support the need for proactive fertility preservation strategies and careful monitoring of reproductive health during treatment. Further research is needed to fully understand the mechanisms of ICI‐induced gonadal toxicity and develop protective interventions.

## Author Contributions

Conceptualisation: David J. Handelsman, Andrea Boutros, Georgina V. Long. Investigation: Georgina V. Long, Alexander M. Menzies, Andrea Boutros. Data curation: Andrea Boutros, Amanda Idan, Sue Sleiman, Feyrous Bacha, Veena Jayadev, David J. Handelsman. Formal analysis: Ting Zhang, David J. Handelsman. Methodology: David J. Handelsman. Project administration: David J. Handelsman, Georgina V. Long. Supervision: David J. Handelsman, Georgina V. Long, Alexander M. Menzies. Writing – original draft: Andrea Boutros, David J. Handelsman. Writing – review and wditing: All co‐authors.

## Conflicts of Interest

G.V.L. is consultant advisor for Agenus, Amgen, Array Biopharma, AstraZeneca, Bayer, BioNTech, Boehringer Ingelheim, Bristol Myers Squibb, Evaxion, GI Innovation, Hexal AG (Sandoz Company), Highlight Therapeutics S.L., Immunocore, Innovent Biologics USA, IOBiotech, Iovance Biotherapeutics, MSD, Novartis, PHMR Ltd, Pierre Fabre, Regeneron, Scancell, SkylineDX B.V.

## Data Availability

Will be provided on reasonable request to the authors.
